# Knowledge of pre-conception health and planned pregnancy among married women in Jinka town, southern Ethiopia and factors influencing knowledge

**DOI:** 10.1371/journal.pone.0268012

**Published:** 2022-05-20

**Authors:** Kassahun Fikadu, Biresaw Wasihun, Osman Yimer

**Affiliations:** 1 Department of Midwifery, College of Medicine and Health Sciences, Arbaminch University, Arba Minch, Ethiopia; 2 Department of Midwifery, College of Medicine and Health Sciences, Debre Birihan University, Debre Birihan, Ethiopia; Flinders University, AUSTRALIA

## Abstract

**Background:**

Optimizing women’s health and knowledge of preconception healthcare before conceiving a pregnancy decreases the risk of adverse pregnancy outcomes. However, preconception health care is one of the missing pillars in the continuum of maternal and child health care in Ethiopia. Therefore, this study aimed to assess knowledge of pre-conception health, its relation to planned pregnancy, parity, family planning use, and education among married women in Southern Ethiopia.

**Methods:**

A community-based cross-sectional study was conducted with 337 married women recruited from March 25 to April 30, 2018 in Jinka town. A simple random sampling technique was employed and the data was collected using a structured questionnaire. Data analysis involved calculating frequencies, percentages, and logistic regression. Associations were assessed using odds ratios and 95% confidence intervals with statistical significance determined at a p-value < 0.05.

**Results:**

The overall women’s preconception health care knowledge score in this study was 55.2%, which is a moderate score. In multivariable analyses, women’s secondary level of education [AOR = 2.3; 95% CI = 1.13–4.87], family planning use [AOR = 2.6, 95% CI = 1.37–4.87], planned pregnancy [AOR = 3.2, 95% CI = 1.35–7.44], Nullyparity [AOR = 21.2; 95% CI = 4.92–91.5], and market trade vendors [AOR = 2.5; 95%CI = 1.06–6.03], were significantly associated with knowledge of preconception health care.

**Conclusion:**

The findings show that women’s knowledge of preconception health care is moderate. Women’s knowledge of preconception health care can be linked to their level of education, use of family planning methods, pregnancy planning, and Nullyparity. Therefore, the government and other key stakeholders need to develop a specific education package that improves women’s knowledge of preconception care and pregnancy planning, taking into account factors such as levels of education and literacy when designing implementation strategies.

## Introduction

Preconception care aims to improve health before conception by facilitating risk screening, health promotion, and effective interventions as part of routine health care [1]. Despite major advances in medical care, poor pregnancy outcomes continue to be a worldwide problem because prenatal care is often provided too late to prevent serious maternal and child health complications. For fetal development, the time leading up to the first prenatal checkup is the most critical. Many women, however, are unable to mitigate risks to their own and their baby’s health since they do not realize they are pregnant until after this important period [2]. Since most women become pregnant before they know that they are pregnant [3], preconception care has a high impact if all reproductive-age couples get involved, whether or not they are contemplating pregnancy [4]. In most low-income settings; however, maternal health care does not start until the pregnancy is well established, and not until more than half of the pregnancy has passed [5]. Others enter into pregnancy without having optimal health status [such as smoking, drinking alcohol, using drugs, and having poor diets and sedentary lifestyles] [1, 6]. Couple often have at least one risky health behavior that predisposes them to an unfavourable pregnancy outcome [7]. There is compelling evidence that maternal and child health issues such as anaemia and obesity, vaccine-preventable diseases such as rubella, mental health issues, personal habits such as tobacco and alcohol use, and exposure to chemicals and radiations all contribute to poor reproductive outcomes [8–11].

Preconception care and pregnancy planning are promising strategies for improving women’s reproductive outcomes, especially for those who have had poor reproductive outcomes in the past [12]. Thus, the preconception period can be viewed as a window of opportunity to improve pregnancy outcomes, as well as children’s and women’s health, at the same time. Women who have no access to preconception healthcare are at an increased risk of unwanted pregnancy, which in turn increases the rate of maternal and infant morbidity and mortality [2]. Research has speculated that about 35% of pregnancies among women with untreated gonococcal infections result in either low birth weight infants or premature deliveries, and up to 10% result in perinatal death [6]. The risk of lower birth weight and preterm delivery often increases in subsequent pregnancies [13]. Further, poor maternal outcomes, such as persistent hypertension [14], preeclampsia, and placental abruption [15], put the baby at risk for pregnancy-related complications. Preconception healthcare awareness, which focuses on addressing women’s pre-existing conditions, can minimize the chance of negative pregnancy outcomes [16]. For example, women who receive preconception care are more aware of potential health issues, which can contribute to a healthier lifestyle later in life [17].

Research suggests preconception health care awareness is low among reproductive-age women in Africa. For example, according to research in Egypt, 62.4% of 660 pregnant women had heard of pre-pregnancy folic acid supplementation, but only 39.2% knew that folic acid supplementation could help avoid congenital abnormalities [18]. Women’s knowledge of seeking preconception health interventions was also reported to be very low in Nigeria and Sudan [19, 20]. In Ethiopia, research findings show that women have low knowledge of preconception health care. For example, a community-based study in Wolayita [21] and Gojjam [22], Ethiopia showed that 53% and about 28% of women had knowledge regarding preconception health care, respectively.

Ethiopia has not yet integrated preconception care into the current healthcare system, and this is a missed opportunity for Ethiopia’s maternity healthcare system to improve the continuum of care and reduce adverse pregnancy outcomes. In a resource-limited setting like Ethiopia, evidence of women’s knowledge of preconception health and associated factors is scarce. Given the importance of epidemiological data in determining the package of services to be provided in routine maternal and child health planning, the purpose of this study was to assess knowledge of pre-conception health, its relation to education, parity, family planning use, and planned pregnancy among married women of childbearing age groups in Jinka, Southern Ethiopia.

## Methods

### Study design and setting

The study was a community-based cross-sectional study conducted from March to April 2018 among married women in the Jinka town administration within the territory of Jinka, 750 km south of Addis Ababa, at a latitude and longitude of 5°47’N 36°34’E/5.783°N 36.567°E. In 2018, the total population was estimated at 30,493, including 15,217 [49.9%] males and 15,276 [50.1%] females. Of the total women of reproductive age, roughly 3147 [44.3%] were reported to be married [23]. The public health infrastructure in the town consists of one general hospital, two health centers, and six health posts where health extension workers serve. Health extension workers are primarily trained to offer community health services in line with the primary health care package. They are responsible for identifying pregnant women within their catchment area, and delivering subsequent antenatal care services. They also keep vital statistics, such as gender, number of children, pregnancy, abortion, death, live births, etc., in the family folder register, including for married women. The study area was selected purposively as it was considered feasible and appropriate to address the study objectives in an urban setting.

### Study participants

The study population consisted of all married women who lived in Jinka town for six months or more. Lastly, individual married women were the study units and self-reported married women who lived in Jinka town were included in this study. Women with hearing problems and critical illnesses were not eligible for the study.

### Sampling technique and procedure

A sample size of 337 was estimated using a single population proportion formula [n = Z^2^ P [P-1]/d2] considering the proportion [P] of women’s level of knowledge of preconception health care to be 27.5% from a study conducted in West Gojjam, Ethiopia [22], Z^2^ [Standard score corresponding to a given confidence interval [CI] of 95%, and the tolerable risk of rejecting the null hypothesis [d = 5%], and a 10% non-response rate. All the kebeles [local administration units] of Jinka town were included. An updated list of households from the 2018 report of each Kebele’s family folder was taken as the sampling frame. A list of married women was identified from the family folder register. The sample size for each kebele was determined proportional to the number of married women per kebele. Using proportional to size allocation, the probability of women being selected is proportional to the size of the overall group of married women of the reproductive age in the kebele, giving a larger proportion of the total group of women participants came from the larger kebeles. A unique code was given to hide the identity and thus, a serial number based on the sequence of registration was taken and frame was formed and fed to the compute to pick the samples randomly. The first married woman’s house was approached by a health extension worker who acted as a guide during the data collection period. A lottery method was applied when more than one candidate was found per household. If an eligible woman in the chosen house was unavailable, the data collector addressed the situation with the individual who was available and set up an appointment with the study subject at a time when she was available. The data collector then returned the next day to see if the women were still at home. Three consecutive days were spent revisiting the woman until she was deemed a non-respondent. Re-visiting helped to reduce the number of non-responses when the interviewee was not present during the data collection day. All women who were approached for participation agreed to take part and were subsequently consented using a short form approved by the Institutional Ethical Review Board.

### Study variables

The dependent variable was women’s knowledge of preconception health care and the independent variables were socio-demographic characteristics, obstetric, and behavioral history such as habit of alcohol intake, smoking, and substance use.

### Survey questionnaire development and pretesting

#### Knowledge

Women’s knowledge of preconception health care was measured using twenty preconception health care questions that we developed for this study. Those who scored above the mean score in the preconception care knowledge questions were considered to have high knowledge. Those who scored less than or equal to the mean score in the preconception care knowledge questions were considered to have low knowledge. Therefore, the scale was dichotomized into high and low knowledge.

### Data collection tool

A face-to-face interview was used to collect data using a pre-tested, structured questionnaire. It consisted of different parts adapted from previously published literature in developing nations [20, 22], and it was modified considering the context and objectives of the study. The questionnaire was divided into sections on socio-demographic characteristics, birth outcomes, chronic illness profiles, general awareness of preconception health care [such as women who have heard about preconception health, their source of information, information on the eligible population, and getting preconception healthcare], as well as questions about pregnancy.

The reliability coefficient was computed using SPSS version 26 window-compatible software and it was 0.86. Each question had one correct response where those who score above the mean of knowledge measuring questions are labeled as women with “High knowledge]. Participants were recruited by five Diploma midwife data collectors and two BSc midwife supervisors who were fluent in the local language and experienced in data collection. They were provided with a two-day training on the objectives of the study, data collection techniques, and informed consent and confidentiality issues. During the data collection period, supportive supervision and panels with the data collectors and supervisors were conducted on a regular basis. Every day, before, during, and after the data collection period, the supervisors checked the questionnaire for clarity and completeness. Throughout the data collection period, the principal investigator was a frontline supervisor.

### Data analysis

Data was first checked for consistency and completeness, missing values, and discordant responses. Then, the data was coded and entered into Epi-info version 7.2 and exported to SPSS software [version 26] for further cleaning and analysis. Descriptive statistics were calculated to determine percentages and frequencies and summary statistics [median and inter quartile range] were used to describe the study population.

Independent variables with a p-value of less than 0.25 in the binary logistic regression analysis were included in the multivariable analysis. The model was adjusted for age. The presence of an association between factors and dependent variables was tested using multiple logistic regression. For the multivariate analysis, a p-value of less than 0.05 was determined as the cut point of a statistically significant association with 95 percent confidence intervals along with the adjusted odds ratio.

### Data quality control

Primarily, the questionnaire was prepared in English and translated into Amharic using experts in both languages. For data analysis, the Amharic version was reverted to the English version to keep the data consistent and clear. Regular meetings were held between data collectors and supervisors to discuss lessons learned and to overcome challenges before the next data collection day. On a regular basis, the principal investigator met with each supervisor. The data collection instrument was pre-tested on 5% of the calculated sample to familiarize the tool for data collectors with the interviewing technique and to ensure consistency. However, neither the location where we conducted the pretesting nor the women on whom the questionnaire was pre-tested were included in the final sample. The Tool Piloting included 18 married women from Karat town and was administered two weeks before the actual data collection began. Data collectors and supervisors were debriefed on the lessons drawn from the pretest and modifications were made for logical order, ambiguity, leading questions, and the addition of details unrelated to the research question the study intended to answer. The instrument’s overall reliability coefficient was 0.86. In addition, content validity was assessed by three independent maternal and child health experts at Arba Minch University.

### Ethical statement

Ethical clearance and a letter of approval to conduct this study were obtained from the institutional board of the College of Medicine and Health Sciences, Arba Minch University and an official letter of cooperation was obtained from Jinka town administration. Written informed consent was obtained from each study participants after explaining the purpose and procedures of the study. The right to withdraw from the study at any time the participants wished to leave was assured and information confidentiality was ensured with coding. Interviews were conducted in a separate area where privacy could be assured. Only the principal investigator had access to the raw data. Parents or guardians of study participants under the age of 18 also gave their written approval.

## Results

### Socio-demographic characteristics

A total of 337 married women were included in this study. The median age of study participants was 27 with an IQR of ±7 [20–34] yrs. Forty-five percent [n = 352] of the study participants were between the age of 25 to 34 years. The majority of participants [45.4%, n = 153] were Orthodox Tewahido religious followers. The majority of the women [55.5%, n = 187] who participated in the study belonged to the Malie ethnic group. The majority [55.5%, n = 187] of women were housewives and 49.6% of respondents had no formal education [n = 167]. In this study, 36.8% of husbands had achieved tertiary education [n = 124]. One hundred thirty-three [39.5%] of the study participants’ husbands were market trade vendors and 214 [63.5%] of the women had access to health-related information through their personal mobile devices [Table 1: Computed by the authors from data collected at the survey].

**Table 1:** Socio-demographic characteristics of child-bearing age women [15–49 years] in Jinka town, Southern Ethiopia, 2018 [n = 337].

Characteristics	Frequency [n = 337]	Percentage [100%]
**Age**		
15–24	115	34.1
25–34	152	45.1
35–49	70	20.8
**Religion**		
Orthodox Tewahido	153	45.4
Muslim	45	13.4
Protestant	139	41.2
**Ethnicity**		
Malie	187	55.5
Oromo	20	5.9
Amhara	102	30.3
Others [Tigray, Wolayita]	28	8.3
**Occupation**		
Housewife	187	55.5
Civil servant	69	20.5
Market trade vendor	60	17.8
Day laborer	21	6.2
**Educational status**		
No formal education	167	49.6
Primary school	76	22.5
Secondary school and above	94	27.9
**Husband educational status**		
No formal education	30	8.9
Primary school	85	25.2
Secondary school	98	29.1
College and above	124	36.8
**Husband occupation**		
Jobless	28	8.3
Market trade vendor	133	39.5
Government employee	87	25.8
Driver	61	18.1
Day laborer	28	8.3
**Means of health information access**		
Radio	78	23.1
Mobile Telephone	214	63.5
TV	45	13.4

### Obstetric and behavioral events

Among the 337 respondents, 73 [21.7%] women were pregnant and 277 [82.2%] of the participants responded that their most recent pregnancy was planned. Two hundred twenty-nine [68%] study participants had been counselled by trained healthcare providers during their last pregnancy. The vast majority of women [79.8%, n = 269] had two to four children. Of the total participants, 26 [7.7%] reported a mistimed pregnancy [pregnancy wanted at a later time]. Forty-five [13.4%] of the study participants had had spontaneous abortions. Women reported various obstetric events such as stillbirth [5.9%], neonatal death [6.8%], preterm birth [2.6%], and congenital anomalies [2.7%]. Moreover, 78.3% of participants [n = 264] had utilized hormonal family planning methods during the last year preceding the study. In our study, 18 [5.3%] women reported alcohol use and 8 [2.4%] reported smoking.

### Women’s chronic health-illness profiles and social behavior characteristics

Fifty-six [16.6%] women had a history of at least one confirmed chronic illness. In this study, women with a history of chronic illness experienced heart disease [4.2%, n = 14], diabetes mellitus [3.9%, n = 13], and others such as asthma, renal disease, pulmonary tuberculosis, anemia, liver disease, and thyroid disease [4.5%, n = 15].

### Married women’s awareness of preconception health care

Among participants, 63.2% of women [n = 213] were aware that preconception services were available. The most frequently mentioned sources of information were health institutions [47.5%] and family/relatives [43.9%]. Two hundred fifty-eight [76.6%] participants agreed that both a woman and her baby could be eligible to receive preconception health care services [Table 2: Computed by the authors from data collected in the survey].

**Table 2:** Women’s awareness of preconception health care in Jinka town, Southern Ethiopia, 2018 [n = 337].

Variables	Frequency [N]	Percentage [%]
Preconception health awareness		
Yes	213	63.2
No	124	36.8
Source of information for preconception health care		
Health institution	160	47.5
Family relatives	148	43.9
Mass media	27	8
Friends	2	0.6
Eligible to get preconception care service		
For baby, only	26	7.7
For the mother, only	24	7.1
For baby and mother	258	76.6
Don’t know	29	8.6
Place to get preconception care		
Home	47	13.9
Health institution	157	46.6
Home and health institution	104	30.9
Don’t know	29	8.6

### Women’s knowledge of preconception health and behavioral risk factors for the fetus

The overall level of knowledge of preconception health care among married women was 55.2%. The results of women’s knowledge of preconception health care components revealed that the habit of cigarette smoking [87.8%], the habit of alcohol consumption [84.0%%], planning pregnancy [84%], and screening for STI and HIV/AIDS [81.6%] were preconception health-related conditions that women have highlighted the most. They also stated that uncontrolled obesity [increase in size and amount of fat cells in the body irrespective of medical or dietary modification used to optimize] [53.4 percent], untreated stress [51.3 percent], and screening for genetic problems [47.5 percent] were the least known conditions related to preconception healthcare [Table 3: Computed by the authors from data collected at the survey].

**Table 3:** Women’s knowledge on preconception health care components in Jinka town, Southern Ethiopia, 2018 [n = 337].

Preconception health knowledge—measured components	Frequency		Percent [%]
Diabetes mellitus should be treated and controlled before conception	Yes	227	67.4
	No	110	32.6
Epilepsy should be treated before conception	Yes	214	63.5
	No	123	36.5
Uncontrolled obesity before conception affects fetal health	Yes	180	53.4
	No	157	46.6
Screening for STI and HIV/AIDS improves fetal life	Yes	275	81.6
	No	62	18.4
Heart disease should be treated and controlled before conception	Yes	215	63.8
	No	122	36.2
Stress before conception affects fetal life	Yes	173	51.3
	No	164	48.7
Screening for a genetic problem lowers poor pregnancy outcomes	Yes	160	47.5
	No	177	52.5
Regular cigarette smoking before conception affects fetal development	Yes	296	87.8
	No	41	12.2
Alcohol drinking before conception leads to poor pregnancy outcome	Yes	283	84.0
	No	54	16.0
Exposure to environmental hazards leads to adverse prenatal outcome	Yes	251	74.5
	No	86	25.5
Habit of Illegal drug intake before conception affects fetal wellbeing	Yes	248	73.6
	No	89	26.4
Female genital mutilation leads to poor pregnancy outcome	Yes	247	73.3
	No	90	26.7
Optimal pre-pregnancy weight prevents adverse pregnancy outcome	Yes	266	78.9
	No	71	21.1
Avoiding cigarette smoking before conception is a must	Yes	260	77.2
	No	77	22.8
Avoiding alcohol consumption before conception is mandatory	Yes	257	76.3
	No	80	23.7
Tetanus vaccination prevents neonatal infection	Yes	270	80.1
	No	67	19.9
Regular medical check-ups helps for a healthy pregnancy outcome	Yes	246	73.0
	No	91	27.0
Folic acid and vitamin supplements lowers fetal malformation	Yes	267	79.2
	No	70	20.8
Creating a healthy environment before conception benefits pregnancy	Yes	254	75.4
	No	83	24.6
Planning pregnancy helps to achieve healthy pregnancy outcomes	Yes	283	84.0
	No	54	16.0

### Factors associated with women’s knowledge of preconception care

Results from the binary logistic regression models for the association between independent variables and women’s preconception health care knowledge, while controlling for age, are presented in Table 4, which is computed by the authors from data collected at the survey.

**Table 4:** Proportion, unadjusted and Adjusted comparison of knowledge of preconception care among married women in Jinka, Southern Ethiopia, 2018 [n = 337].

Variables		Women’s Knowledge of preconception health care		Crude Odds ratio [95%CI]	Adjusted Odds ratio [95%CI]	*P-value*
		Low[n = 151]	High[n = 186]			
**Women’s occupation**	Housewife	106 [56.7%]	81 [43.3%]	Ref	Ref	
	Civil servant	27 [39.1%]	42 [60.9%]	2.0 [1.16–3.58]	1.4 [0.74–2.58]	0.31
	Market trade vendor	12 [20%]	48 [80%]	5.2 [2.61–10.5]	2.5 [1.06–6.03]	0.04
	Day laborer	6 [28.6%]	15 [71.4%]	3.3[1.22–8.80]	2.4[0.82–7.06]	0.11
**Women’s Education**	No formal education	100 [66.2%]	67 [36.0%]	Ref	Ref	
	Primary education	28 [18.5%]	48 [25.8%]	2.6 [1.46–4.48]	1.7 [0.92–3.18]	0.09
	Secondary education and above	23 [15.3%]	71 [38.2%]	4.6 [2.62–8.09]	2.3 [1.13–4.87]	0.02
**Parity**	Nullyparity	8 [5.3%]	19 [10.2%]	8.4 [2.79–25.6]	21.2[4.9–91.5]	0.00
	Primiparity	24 [15.9%]	54 [29.0%]	8.0 [3.31–19.3]	4.9[1.86–12.9]	0.00
	Multiparity	87 [57.6%]	104[55.9%]	4.3[1.92–9.39]	3.0[1.29–7.06]	0.01
	Grand-multiparity	32 [21.2%]	9[4.9%]	Ref	Ref	
**History of Family planning use**	No	48 [31.8%]	25 [13.4%]	Ref	Ref	
	Yes	103 [68.2%]	161 [86.6%]	3.0 [1.74–5.17]	2.6 [1.4–4.78]	0.00
**Pregnancy characteristic**	Planned	121 [80.1%]	156 [83.9%]	2.6 [1.2–5.5]	3.3 [1.41–7.58]	0.01
	Nulliparous	8 [5.3%]	19 [10.2%]	4.8 [1.6–14.2]	-	
	Mis-timed	22 [14.6%]	11 [5.9%]	Ref	Ref	

Women whose occupation is trade vendor were 2.5 times more likely to know preconception health than women whose occupation is housewife [AOR: 5.2; 95% CI: 1.06, 6.03]. The likelihood of having high preconception health knowledge was higher in women whose education level was secondary education and above [AOR = 2.3; 95% CI = 1.13–4.87] compared to those with no formal educational status. The odds of having high preconception healthcare knowledge were increased in nulliparous [AOR = 21.2; 95% CI = 4.92–91.5], primiparous [AOR = 4.9; 95% CI = 1.86–12.9], and multiparous [AOR = 3.0; 95% CI = 1.29–7.06] women than their grand-multiparous [a woman who has had more than five births [live or stillborn] at greater than 28 weeks of gestation] counterparts. Further, women who had used family planning methods had higher odds of high preconception health knowledge [AOR = 2.6, 95%CI = 1.37–4.87]. Lastly, women who had planned pregnancies had an increased likelihood of high preconception health knowledge [AOR = 3.3, 95%CI = 1.41–7.58].

## Discussion

In this study, the overall study participants’ knowledge of pre-conception health care was found to be 55.2%. This findings are similar to results found in other parts of Ethiopia [53%] [21], Malaysia [51.9%] [35], and Qatar [53.7%] [36] but higher than a study conducted elsewhere in Ethiopia [27.5%] [22], and other countries such as Saudi Arabia [42.8%] [24], Nigeria [20.6%] [25], India [6%] [26], Egypt [22%] [27], South-Eastern Nigeria [43.1%] [28], and Sudan [11.1%] [19]. This may be due to time variation; time may enhance women’s ability to make a positive change, which leads to a better understanding of preconception healthcare. Further, in the case of the Sudanese and Saudi Arabian studies, a single component of preconception health care was addressed. In Gojjam, the difference may be due to differences in educational attainment, since more women in this study had completed primary education. In general, it may also be due to variation in study settings, study participants, and health-care systems of the countries. The participation of rural women may also be a source of heterogeneity, while the current study focuses on women living in urban areas.

Preconception knowledge is lower in this sample than in middle and high income countries such as Jordan [85%] [29], Iran [68.8%] [30], China [90%] [31], Canada [70%] [32], and the United States [76%] [33]. The moderate level of knowledge of preconception health in this study may be due to the fact that women who live in middle to high-income countries have greater access to information, internet access, and better media coverage. On the other hand, preconception and pregnancy planning information may not be available to them, in their language, even though most do have personal mobile devices. Indeed, preconception health care is not part of the routine healthcare system of Ethiopia. In developed regions, there is a large health-care infrastructure, a high socioeconomic status index, and widespread media coverage of preconception health. In Ethiopia, the health care system has paid insufficient attention to Preconception Care [PCC] implementation across the country, resulting in a lack of preconception clinics at the health institution level and a low commitment of health care workers due to a high case flow of clients. In fact, the presence of specialty clinics designed to give maternal care given by a female birth attendant might enhance women’s interest in preconception health care.

According to the findings of this study, trade vendors were more likely to be aware of preconception health. The current study’s findings are consistent with those of a study conducted in Ethiopia [34], which found that women’s knowledge level is determined by their work. It is possible because trade vendors typically interact with a diverse range of people, which allows them to gain better access to health information and, as a result, develop good communication skills.

In this research, knowledge of preconception health care increased with increasing levels of education. This result is consistent with different studies conducted in Ethiopia [22], China [35], Nigeria [25], Sudan [19], and Iran [30]. An educated woman likely has increased access to preconception care information. The higher the women’s education level, the better she understands preconception health care services and has a favorable attitude towards preconception health services [36]. It implies that women with a higher level of education may have better access to and understanding of information based on its relevance for the intended purpose, which enhances the level of knowledge and intention to be prepared for planned pregnancy [37].

This study showed that nulliparous women have an increased probability of knowing about preconception health care. For a married woman because of desire to have baby, she may aspire to know more about conception before getting pregnant. Information seeking behavior will thus be enhanced. This could be achieved through reading, asking questions, and watching videos where she could have the opportunity to be aware of preconception health care [38, 39]. In this regard, having access to health information from health facilities may contribute to improving participants’ preconception health knowledge [40]. The current study finding is in agreement with different studies conducted in low and middle income countries [19, 41, 42]. Further, a woman who has an intention to get pregnant may seek health care where she receives medical advice and counseling [41]. Poor media coverage, as well as the recent introduction of pre-service preconception health courses into primary healthcare curricula in Ethiopia, may have contributed to Nulliparous women having a better understanding of preconception care [43]. Furthermore, because the women believe they are unfamiliar with conception and its changes [44], it is likely that nulliparous women will seek out additional information, allowing them to learn more about preconception health. In addition, women who used family planning methods had higher pre-conception knowledge than those who did not. This finding is consistent with studies conducted in Ethiopia [22] and Sudan [19]. Since planning a pregnancy is a vital component of preconception health [8], women who have access to family planning services might know about the risk of an unplanned pregnancy. It may be because pregnancy counseling and family planning services are integrated where family planning women have an opportunity to be aware of preconception health care. This could be due to a family planning service, which aims to improve people’s health before they have a planned pregnancy [8]. Women who postpone their pregnancy have an increased need for preconception counseling [45]. This study also indicated that planning pregnancy is associated with knowledge of preconception care. Similarly, studies done in Ethiopia [41], Brazil [46], China [31], and Iran [47] revealed that women who wanted or supported a pregnancy were more likely to know about preconception health. Women who are planning a pregnancy are more likely to seek medical advice and counseling before becoming pregnant.

### Limitations of this study

One of the limitations of this study is that due to self-reporting of data, social desirability and recall biases may affect the responses. However, this was reduced by conducting one-on-one interviews at comfortable place in their home, and also limiting the reporting time to 12 months to reduce recall bias. Moreover, the data collectors received thorough training. On the other hand, this study did not address an obstetric parameter such as a delivery week, delivery mode, and birth weight. Preconception, and health related information access via personal mobile devices does not asked, the study addressed general information. Future research should, therefore, consider healthcare providers and institutional-related factors. Finally, the cross-sectional nature of the study limits the ability to draw causal conclusions between the variables.

## Conclusion

This study found that more than half of the women in the study has some knowledge of preconception healthcare. A woman’s educational status, parity, pregnancy planning, and family planning use were significantly associated with knowledge of preconception health care. Understanding the perspectives of reproductive-aged women and scaling up health education with a focus on women with poor educational status, unplanned pregnancy, and no usage of family planning are crucial.

As a result, the collaboration of various stakeholders, such as local health offices, non-governmental organizations, media personnel, and health care providers, is critical in order to work toward the establishment of preconception care strategies that can address all components of preconception care.

## Supporting information

S1 File (SAV)

## Abbreviations

CI: Confidence Interval
LMIC: Low and Middle-Income country
PCHC: Preconception health care
WHO: World health organization
